# Supplementary material for: Oncogenic context shapes the fitness landscape of tumor suppression
Source: Nat Commun. 2023 Oct 12;14:6422. doi: 10.1038/s41467-023-42156-y (PMC10570323; doi:10.1038/s41467-023-42156-y)
Supplement: Supplementary file 1 — Supplementary Information [file 41467_2023_42156_MOESM1_ESM.pdf]

SUPPLEMENTARY INFORMATION FOR:

Oncogenic context shapes the fitness landscape of tumor suppression

Lily M. Blair[1*], Joseph M. Juan[1*], Lafia Sebastian[1], Vy B. Tran[1], Wensheng Nie[1], Gregory D. Wall[1], Mehmet Gerceker[1], Ian K. Lai[1], Edwin A. Apilado[1], Gabriel Grenot[1], David Amar[1,2,3], Giorgia Foggetti[4], Mariana Do Carmo[5], Zeynep Ugur[4], Debbie Deng[6], Alex Chenchik[6], Maria Paz Zafra[7,8,9], Lukas E. Dow[7,10,11], Katerina Politi[4,5,12], Jonathan J. MacQuitty[1], Dmitri A. Petrov[13,14], Monte M. Winslow[15,16], Michael J. Rosen[1,17#], and Ian P. Winters[1,18#]

AUTHOR AFFILIATIONS

[1] D2G Oncology, Mountain View, CA, USA.

[2] Department of Biomedical Data Science, Stanford University School of Medicine, Stanford, CA, USA.

[3] Department of Cardiovascular Medicine and the Cardiovascular Institute, Stanford University School of Medicine, Stanford, CA, USA.

[4] Yale Cancer Center, Yale School of Medicine, New Haven, CT, USA.

[5] Department of Pathology, Yale School of Medicine, New Haven, CT, USA.

[6] Cellecta, Mountain View, CA, USA.

[7] Sandra and Edward Meyer Cancer Center, Department of Medicine, Weill Cornell Medicine, New York, NY, USA.

[8] Excellence Research Unit "Modeling Nature" (MNat), University of Granada, E-18016 Granada, Spain.

[9] Instituto de Investigación Biosanitaria de Granada (ibs.GRANADA), E-18071 Granada, Spain.

[10] Weill Cornell Graduate School of Medical Sciences, Weill Cornell Medicine, New York, NY, USA.

[11] Department of Medicine, Weill Cornell Medicine, New York, NY, USA.

[12] Section of Medical Oncology, Department of Internal Medicine, Yale School of Medicine, New Haven, CT, USA.

[13] Department of Biology, Stanford University, Stanford, CA, USA.

[14] Chan Zuckerberg BioHub, San Francisco, CA USA.

[15] Department of Genetics, Stanford University School of Medicine, Stanford, CA, USA.

[16] Department of Pathology, Stanford University School of Medicine, Stanford, CA, USA.

[17] mike@d2g-oncology.com

[18] ian@d2g-oncology.com

[*]These authors contributed equally

[#]Corresponding authors

**AACR GENIE (N=13,721 LUAD patients)**															
**Mutation(s):**	***KRAS***	***BRAF***	***EGFR***	***KRAS G12C***	***KRAS G12D***	***BRAF V600E***	***EGFR L858R***	***KRAS codon 12***	***KRAS codon 13***	***KRAS codon 61***	***BRAF Class 1***	***BRAF Class 2***	***BRAF Class 3***	***EGFR exon 19 deletion***	***EGFR exon 20 insertion***
Number of samples:	4869	892	3756	1941	686	253	1121	4130	354	315	256	208	194	1564	286
Fraction of samples:	0.35	0.06	0.27	0.14	0.05	0.02	0.08	0.30	0.03	0.02	0.02	0.02	0.01	0.11	0.02
**TCGA (N=562 LUAD patients)**															
**Mutation(s):**	***KRAS***	***BRAF***	***EGFR***	***KRAS G12C***	***KRAS G12D***	***BRAF V600E***	***EGFR L858R***	***KRAS codon 12***	***KRAS codon 13***	***KRAS codon 61***	***BRAF Class 1***	***BRAF Class 2***	***BRAF Class 3***	***EGFR exon 19 deletion***	***EGFR exon 20 insertion***
Number of samples:	168	42	75	70	20	9	23	149	10	4	9	8	13	23	3
Fraction of samples:	0.30	0.07	0.13	0.12	0.04	0.02	0.04	0.27	0.02	0.01	0.02	0.01	0.02	0.04	0.01

Supplementary Table 1. Frequency of oncogenic KRAS, EGFR, and BRAF mutations in LUAD.

	KRAS G12C	KRAS G12D	EGFR L858R	BRAF V600E
Apc	New			
Arid2	New		New	New
Atm	New			New
Atrx	New		New	New
Brca2	New		New	New
Cdkn2a	New			
Cmtr2	New		New	New
Fbxw7	New		New	New
Kdm6a	New		New	New
Keap1	New			New
Kmt2d	New		New	New
$Kras^{WT}$	New		New	New
Lkb1	New			
Mga	New		New	New
Msh2	New		New	New
Nf1	New		New	New
Nf2	New		New	New
Palb2	New	New	New	New
Pten	New		New	New
Ptprd	New		New	New
Rb1	New			New
Rbm10	New			
Rnf43	New		New	New
Setd2	New			New
Smad4	New			New
Stag2	New		New	New
Tsc1	New		New	New
p53	New			
Total new oncogene by tumor suppressor genetic interactions investigated in the current manuscript	**28**	**1**	**18**	**23**

Supplementary Figure 1. Extent of novel oncogene by tumor suppressor genetic interactions described in the current manuscript. Previous studies have investigated the impact of inactivating putative tumor suppressor genes on lung tumor growth in similar genetically engineered models. "New" indicates that this oncogene by tumor suppressor combination has not been previously investigated. The number of new oncogene-by-tumor suppressor combinations analyzed in this manuscript is indicated at the bottom of each column (70 out of the 112 possible combinations have not been investigated previously). Many of the genes investigated in the oncogenic KRAS G12D-driven model have been investigated using conventional tumor suppressor floxed alleles as well as using Tuba-seq[1, 2]. All tumor suppressors analyzed in the model of EGFR-driven lung cancer were performed using Tuba-seq[3]. The tumor suppressors studied in the model of BRAF-driven lung cancer have all been analyzed using conventional floxed models, hence there has been no systematic cross comparison of tumor suppressor effects within that model. See Supplementary Fig. 12 for a summary of existing data that compared tumor suppressor effects across (rather than within) genetically engineered lung cancer mouse models.

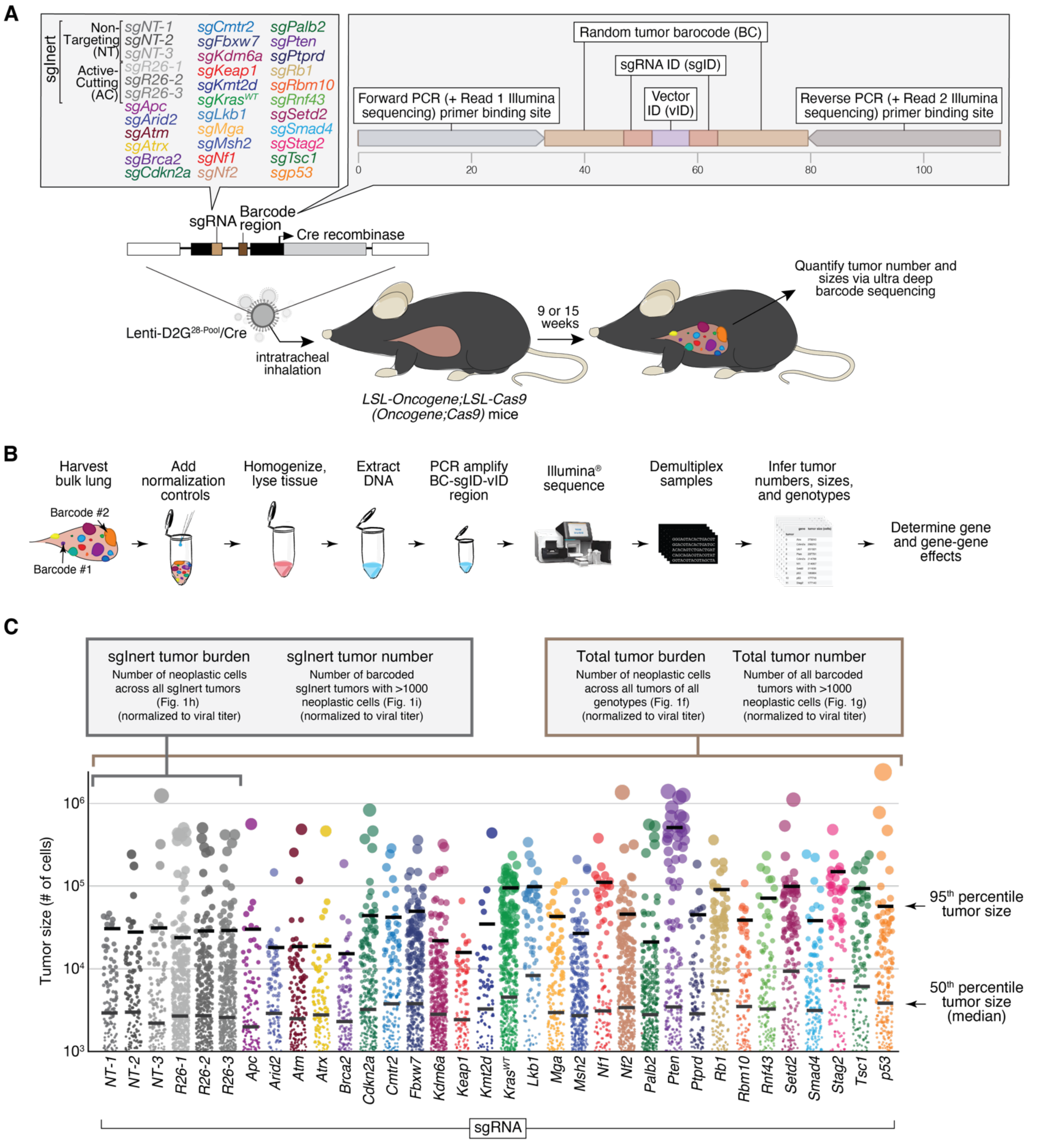

Supplementary Figure 2. Integration of CRISPR/Cas9-mediated gene editing and tumor barcoding into genetically engineered mouse models enables the generation of tumors with diverse combinations of activated oncogenes and inactivated tumor suppressors within individual mice. **A,** General experimental schematic depicting the composition of the pool of barcoded Lenti-sgRNA/Cre vectors (Lenti-D2G$^{28\text{-}Pool}$/Cre) and composition of the three-component barcode. **B,** Overview of sample processing starting from bulk lung. The addition of barcoded normalization controls at a known number allows the number of neoplastic cells in each tumor to be calculated (see Methods). **C,** Jitter plot of all barcoded tumors detected in one *G12D;Cas9* mouse with Lenti-D2G$^{28\text{-}Pool}$/Cre initiated tumors. Each dot represents a tumor, and the dot size is scaled to tumor size. The size of the 95th percentile and 50th percentile (median) tumor within the size distribution of tumors with each Lenti-sgRNA/Cre vector is indicated. Metrics of total tumor burden, total tumor number, sgInert tumor number, and sgInert tumor burden are shown.

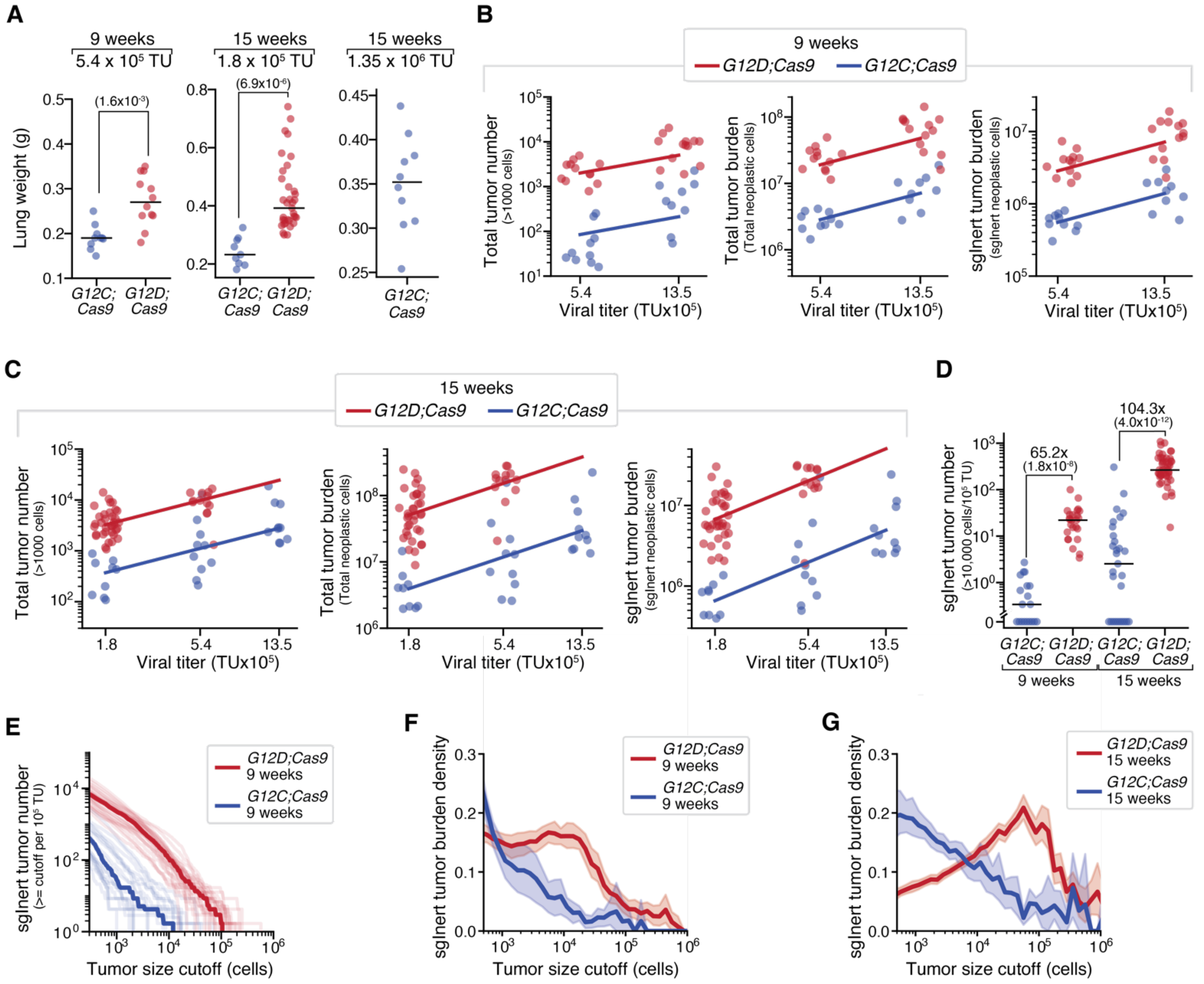

Supplementary Figure 3. KRAS G12C consistently generates lower tumor burden than KRAS G12D. **A,** Lung weights of mice transduced with the indicated titers of Lenti-D2G$^{28\text{-Pool}}$/Cre. Genotype and time post-tumor initiation is indicated. Each dot represents a mouse, and the bar is the median. Fold difference between medians and significance calculated using a Wilcoxon rank sum test (p-values < number in parentheses) is shown. **B,** Total tumor number, total tumor burden, and sgInert tumor burden number of neoplastic cells in mice 9 weeks post-tumor initiation with the indicated titer of virus. Mouse genotypes are indicated. Each dot represents a mouse, and the bar is the median. Lines represent a linear fit through the origin, showing the increases in tumor number and burden are linear with titer. **C,** Total tumor number, total tumor burden, and sgInert tumor burden number of neoplastic cells in mice 15 weeks post-tumor initiation with the indicated titer of virus. Mouse genotypes are indicated. Each dot represents a mouse, and the bar is the median. Lines represent a linear fit through the origin, showing the increases in tumor number and burden are linear with titer. **D,** Number of large sgInert tumors greater than 10,000 cells in size, normalized to viral titer. Mouse genotypes and time-points are indicated. Each dot represents a mouse, and the bar is the median. Fold difference and significance calculated using a Wilcoxon rank sum test (p-values < number in brackets) are shown. **E,** Number of tumors at or above the tumor size cutoff on the x-axis in *G12D;Cas9* and *G12C;Cas9* at 9 weeks post-tumor initiation. Each transparent line represents a mouse, and the solid line is the median. **F** and **G,** The density function of sgInert tumor burden as a function of log(tumor size) 9 weeks (**F**) and 15 weeks (**G**) post-tumor initiation.

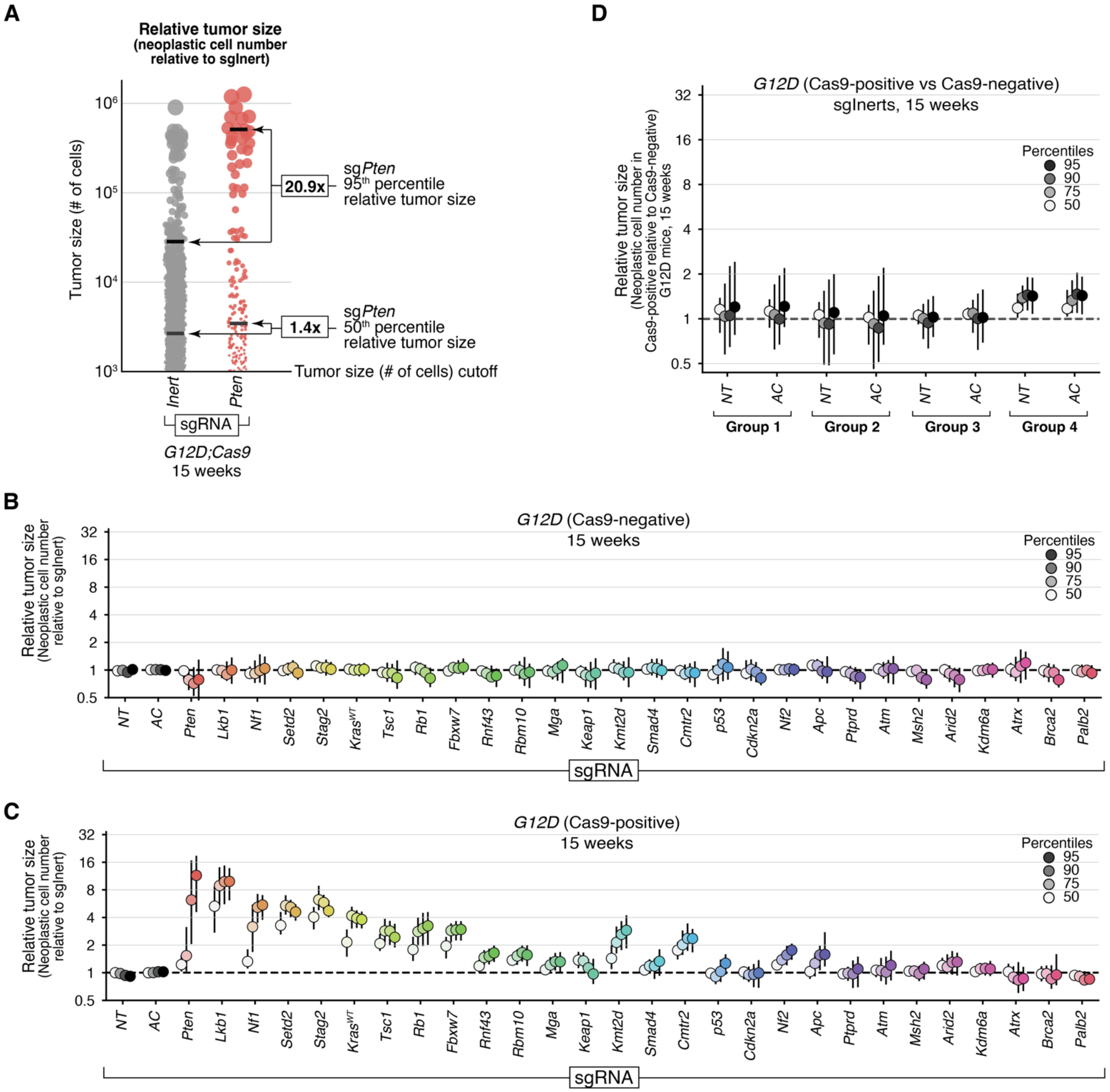

Supplementary Figure 4. Relative tumor size percentiles enable quantification of effects of Cas9-mediated tumor suppressor inactivation on tumor growth. **A,** Schematic representation of the calculation of relative tumor size (neoplastic cell number relative to sgInert) using simulated sgInert and sgPten tumor size distributions as an example. For all tumors above a defined tumor size (# of neoplastic cells) cutoff (1000 cell cutoff is shown), the size (# of neoplastic cells) of the 50th percentile (median) tumor within the sgPten tumor size distribution divided by the size of the 50th percentile tumor within the sgInert tumor size distribution is used to calculate the sgPten 50th percentile relative tumor size. Relative tumor size can be calculated using the same procedure for any matching percentile (the 95th percentile is also shown) tumor in the sg[TumorSuppressor] versus sgInert tumor size distributions. Note that this data from KrasG12D:Cas9 mice is from a repeat study (corresponding to Group 3 in Supplementary Fig. 5a,b) which is distinct from that shown in Fig. 2a. **B,C,** Relative size (neoplastic cells) of the tumor at the indicated percentiles of the tumor size distributions for barcoded Lenti-sgRNA/Cre vectors targeting each gene, relative to the size of the sgInert tumor at the same percentile in G12D mice (b) and G12D;Cas9 mice. **D,** Relative size (neoplastic cells) of the tumor at the indicated percentiles of the tumor size distributions for barcoded sgInert tumors in G12D;Cas9 mice (Cas9Positive) relative to the size of the sgInert tumor at the same percentile in G12D mice (Cas9-negative). The study groups used here correspond to the groups with the same labels in Supplementary Fig. 5a,b. All groups from that list that had a corresponding Cas9-negative group were included.

A

G12D;Cas9

		Mouse number	Viral titer (TU/mouse)
	Group 1	41	180,000
	Group 2	15	180,000
	Group 3	33	180,000
	Group 4	10	180,000
	Group 5	24	180,000
	Group 6	24	180,000
	Group 7	25	180,000
	Group 8	23	180,000
Main Exp.	Group 9	13	540,000
Main Exp.	Group 10	17	180,000
Main Exp.	Group 11	18	180,000

G12C;Cas9

		Mouse number	Viral titer (TU/mouse)
Main Exp.	Group 1	9	180,000
Main Exp.	Group 2	10	540,000
Main Exp.	Group 3	10	1,350,000
Main Exp.	Group 4	18	1,800,000

B

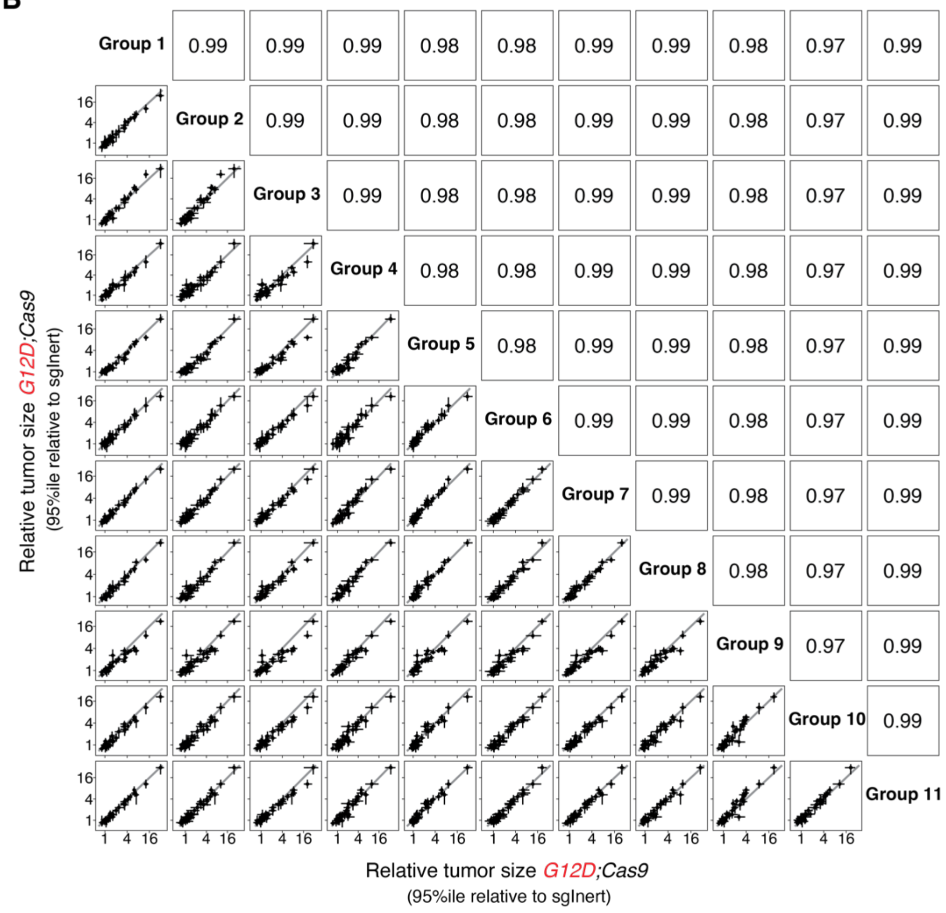

C

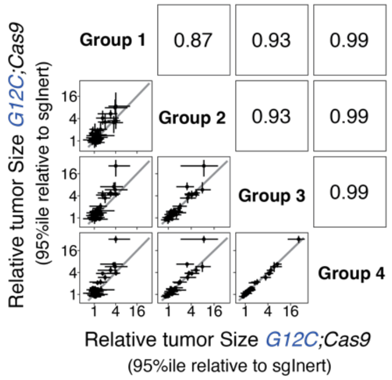

D

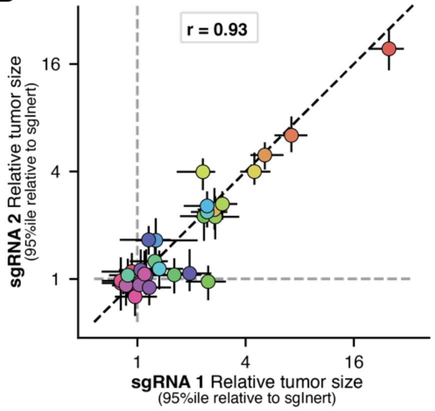

E

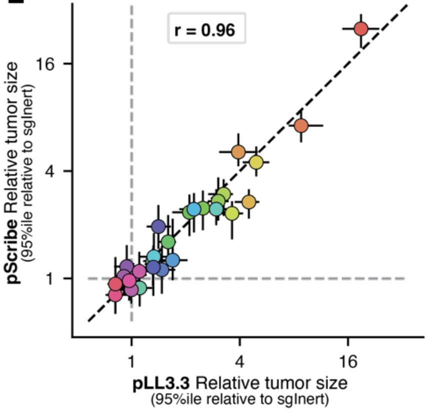

Supplementary Figure 5. Tumor suppressor effects are highly reproducible across studies and sgRNAs targeting the same gene. **A,** Replicate studies and groups used to assess the reproducibility of the impact of inactivating tumor suppressors on lung cancer growth in *G12D;Cas9* (**top**) and *G12C;Cas9* (**bottom**) mice. Mouse number and viral titers are shown for each group. **B** and **C.** 95th percentile Relative tumor sizes (relative to sgInert) for each of the 11 *G12D;Cas9* study groups (**B**) and 4 *G12C;Cas9* study groups (**C**). Each point represents tumors initiated with the same Lenti-sgRNA/Cre vector and the bars are the 95th percent confidence intervals. Grey line indicates equal effect. Pearson r is indicated. Note that 3 pairs of groups (1 and 2; 6 and 7; 10 and 11) in panel "**A**" are replicate groups of mice within the same study, while all other listed Groups are from distinct, independent studies initiated at different times**. D.** 95th percentile relative tumor sizes (relative to sgInert) for 2 distinct sgRNAs targeting the same gene in 34 *G12D;Cas9* mice. Guide 1 (x-axis) represents the sgRNA used in the main experiment. sgRNAs performance was tested using a different, but equivalent lentivector than was used in the main experiment (see **E** for comparison). Each point represents tumors initiated with the same Lenti-sgRNA/Cre vector and the bars are the 95th percent confidence intervals. Black dotted line indicates equal effect. Pearson r is indicated. **E.** 95th percentile relative tumor sizes (relative to sgInert) for the sgRNA used in the main experiment on 2 different plasmid backbones in *G12D;Cas9* mice. pLL3.3 is the backbone used in the main experiment. Each point represents the tumors initiated with one Lenti-sgRNA/Cre vector and the bars are the 95th percent confidence intervals. Black dotted line indicates equal effect. Pearson r is indicated.

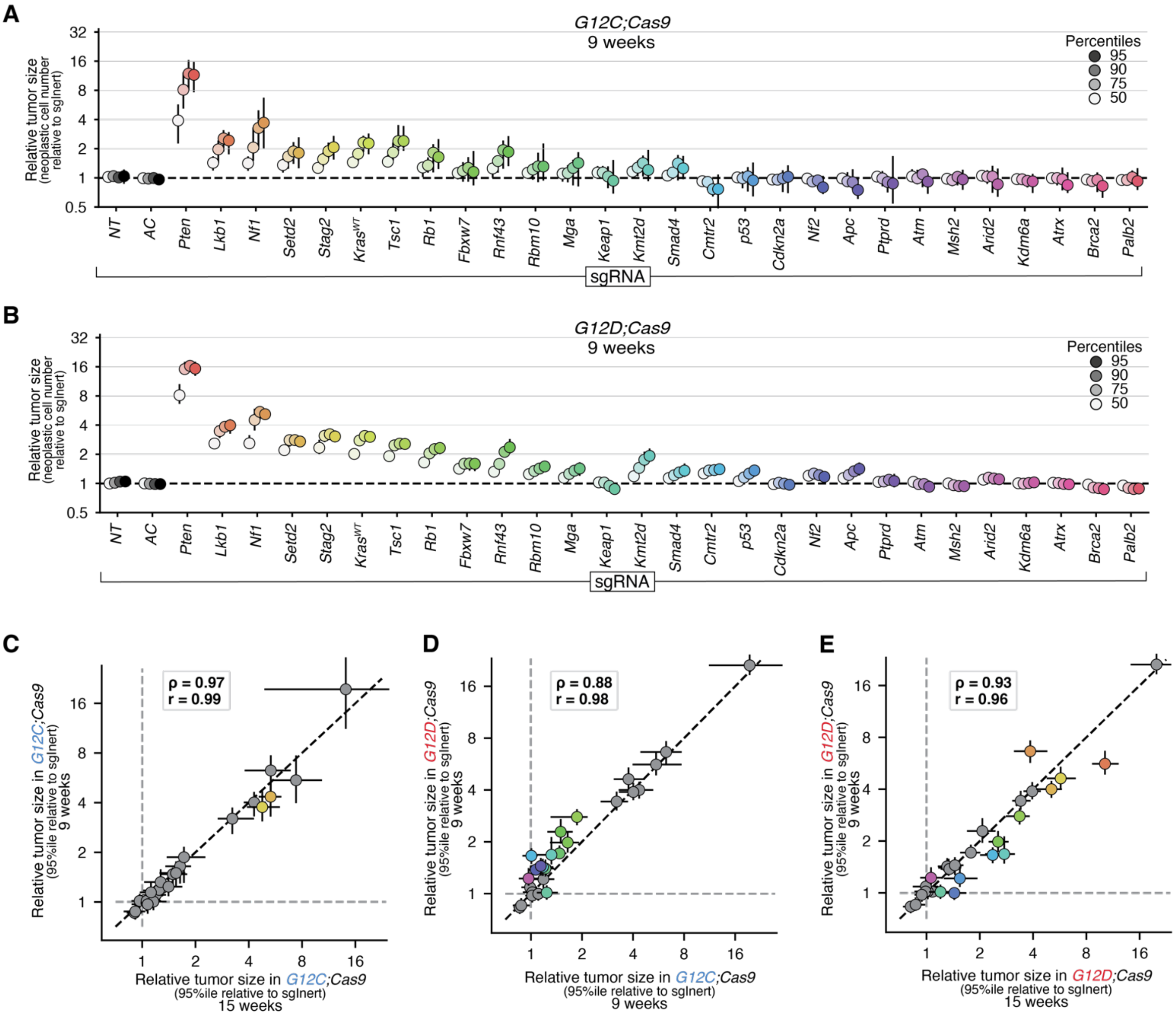

Supplementary Figure 6. Tumor suppressor effects are reproducible across different timepoints and studies. **A** and **B,** Relative size (neoplastic cells) of the tumor at the indicated percentiles of the tumor size distributions for barcoded Lenti-sgRNA/Cre vectors targeting each gene, relative to the size of the sgInert tumor at the same percentile, in *G12C;Cas9* mice (**A**) and *G12D;Cas9* mice (**B**) at 9 weeks after tumor initiation. 95% confidence intervals are shown. **C-E**. Relative size of the tumor at the 95th percentile of the tumor size distributions in the indicated genotypes of mice at the indicated times after tumor initiation. Each dot represents the tumors initiated from one Lenti-sgRNA/Cre vector and the bars are the 95th percent confidence intervals. Genes where the 95% CI excluded no effect in both of the two groups are shown in color. Black dotted line indicates equal effect. Spearman rank-order correlation (ρ) and Pearson correlation (r) are indicated.

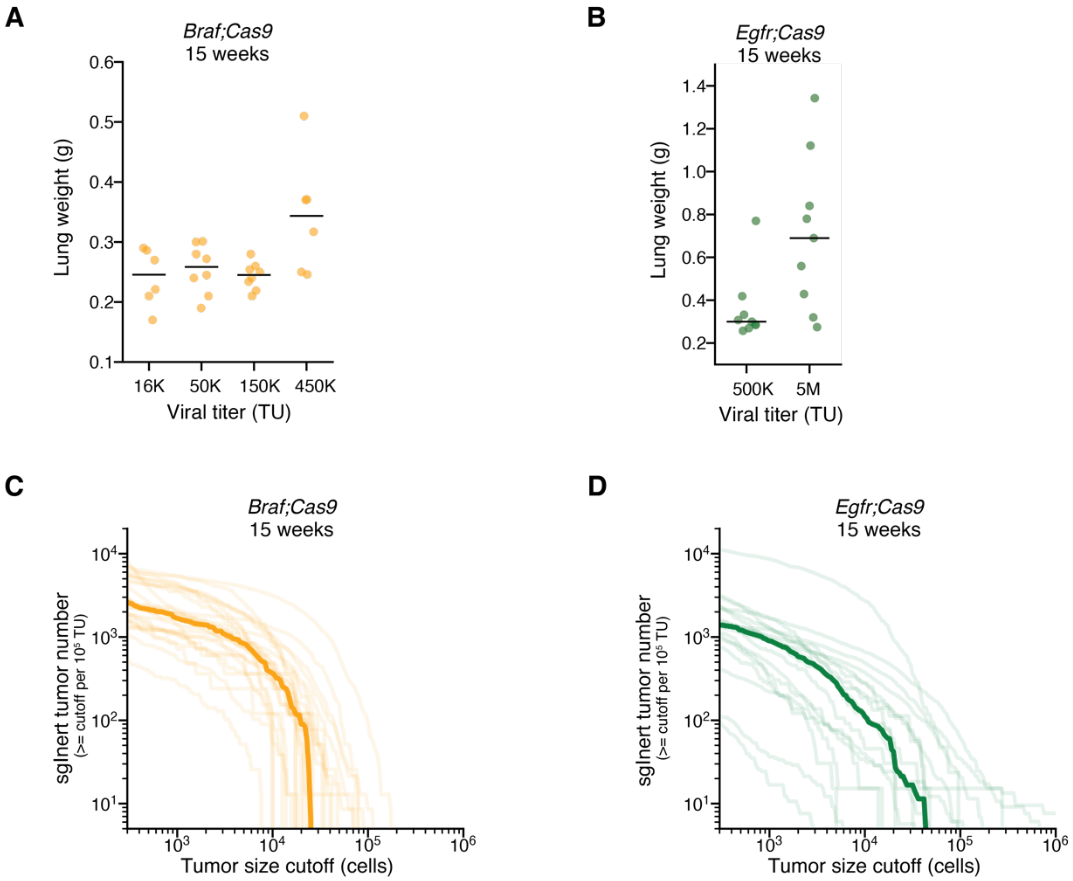

Supplementary Figure 7. Oncogenic BRAF and EGFR drive the formation of lung tumors with differently shaped size distributions *in vivo.* **A** and **B,** Lung weights of mice transduced with the indicated titers of Lenti-D2G28-Pool/Cre in *Braf;Cas9* (**A**) and *Egfr;Cas9* (**B**) at 15 weeks post-tumor initiation. Each dot represents a mouse, and the bar is the median. **C** and **D.** Number of tumors at or above the tumor size cutoff on the x-axis in *Braf;Cas9* (**C**) and *Egfr;Cas9* (**D**) at 15 weeks post-tumor initiation. Each transparent line represents a mouse, and the solid line is the median.

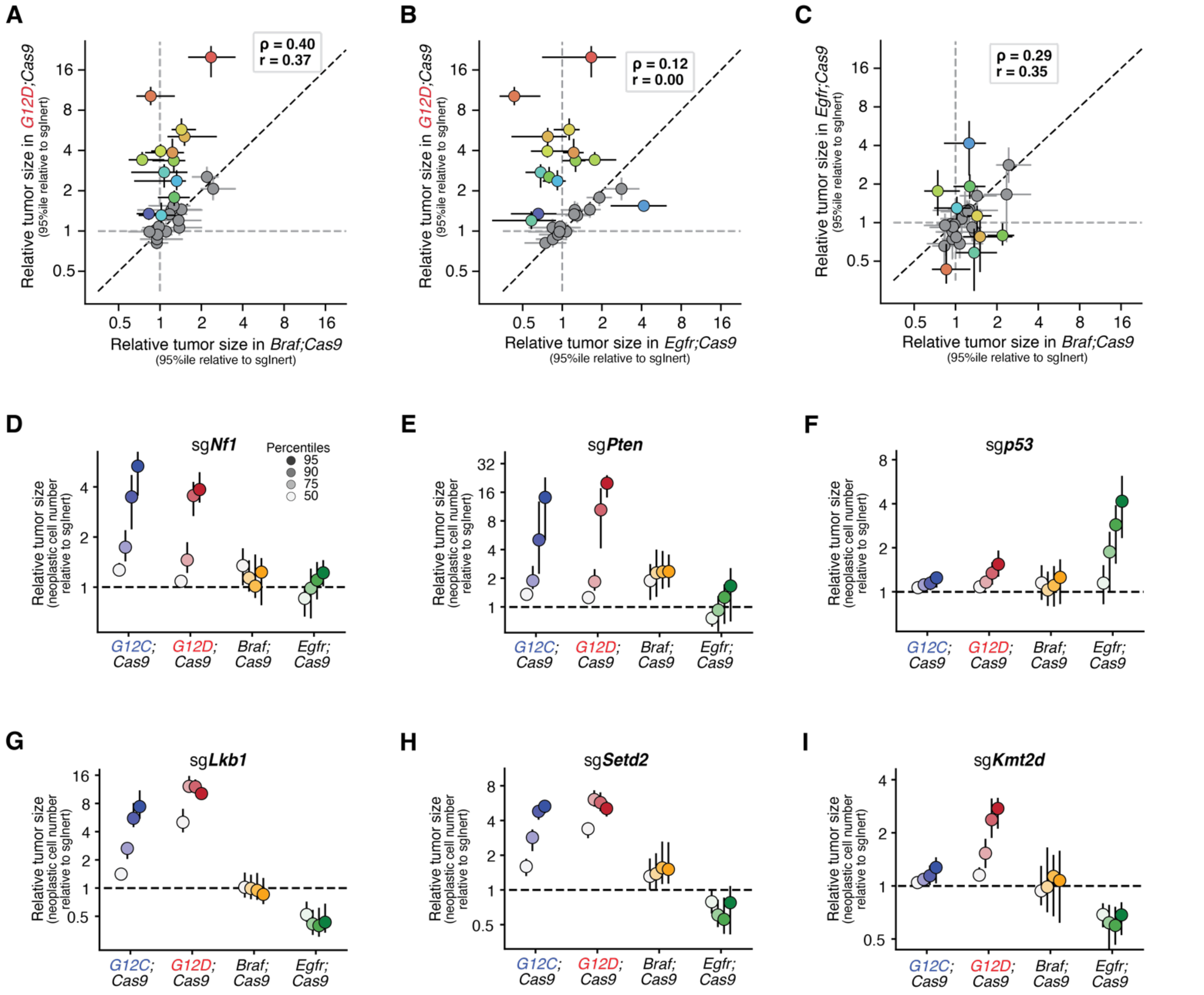

Supplementary Figure 8. Effect of tumor suppressor inactivation is dependent on oncogenic context. **A-C,** Relative size of the tumor at the 95th percentile of the tumor size distributions in the indicated genotypes of mice at the indicated times after tumor initiation. Each dot represents the tumors initiated from one Lenti-sgRNA/Cre vector and the bars are the 95th percent confidence intervals. Genes where the 95% CI excluded no effect are shown in color. Black dotted line indicates equal effect. Spearman rank-order correlation (ρ) and Pearson correlation (r) are indicated. **D-I,** Relative size of the tumor at the indicated percentiles (legend in **D**) of the tumor size distributions for Lenti-sgRNA/Cre vectors targeting *Nf1* (**D**), *Pten* (**E**), *p53* (**F**), *Lkb1* (**G**), *Setd2* (**H**), and *Kmt2d* (**I**) in tumors in the indicated genotypes of mice.

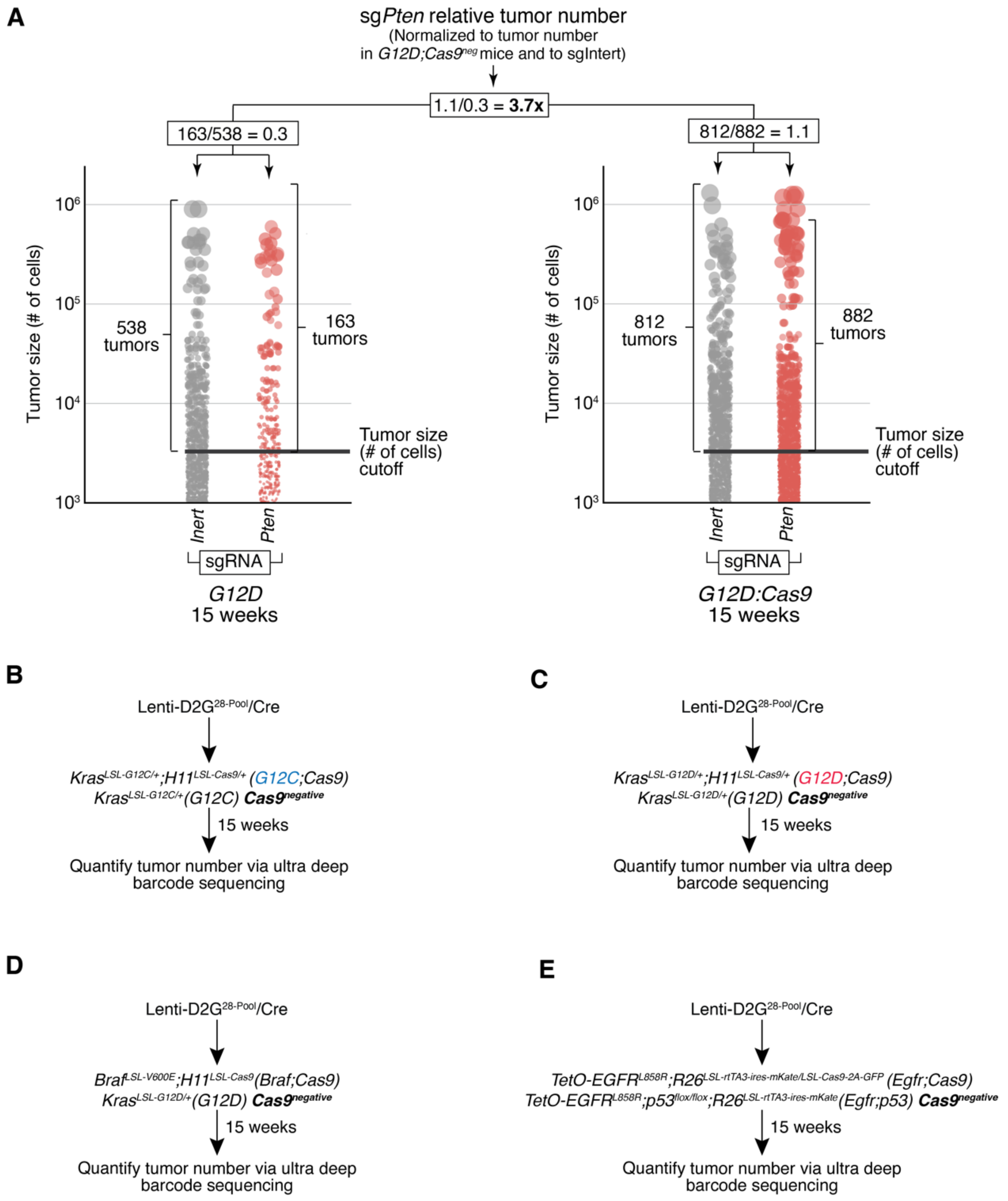

Supplementary Figure 9. Relative tumor number measurements enable quantification of effects of Cas9-mediated tumor suppressor inactivation on tumor number. **A,** Schematic representation of the calculation of relative tumor number (normalized to tumor number in *Cas9*^{negative} mice and to sgInert) using simulated sgInert and sg*Pten* tumor size distributions as an example. To calculate the sg*Pten* relative tumor number: for all tumors above the tumor size cutoff, the ratio of the number of sg*Pten* tumors to sgInert tumors in the *G12D;Cas9* mice is divided by the ratio of the number of sg*Pten* tumors to sgInert tumors in the *G12D* mice. Note that the ratio of sg*Pten* tumors to sgInert tumors in the *G12D* mice reflects the relative titers of Lenti-sg*Pten*/Cre to Lenti-sgInert/Cre in the virus pool, not a biological effect of *Pten* inactivation, as all guides are inert in *Cas9*^{negative} mice. **B-E,** Description of the mouse genotypes used to calculate relative tumor numbers for *G12C;Cas9* (**B**), *G12D;Cas9* (**C**), *Braf;Cas9* (**D**), and *Egfr;Cas9* (**E**) in **Fig. 5A-D**.

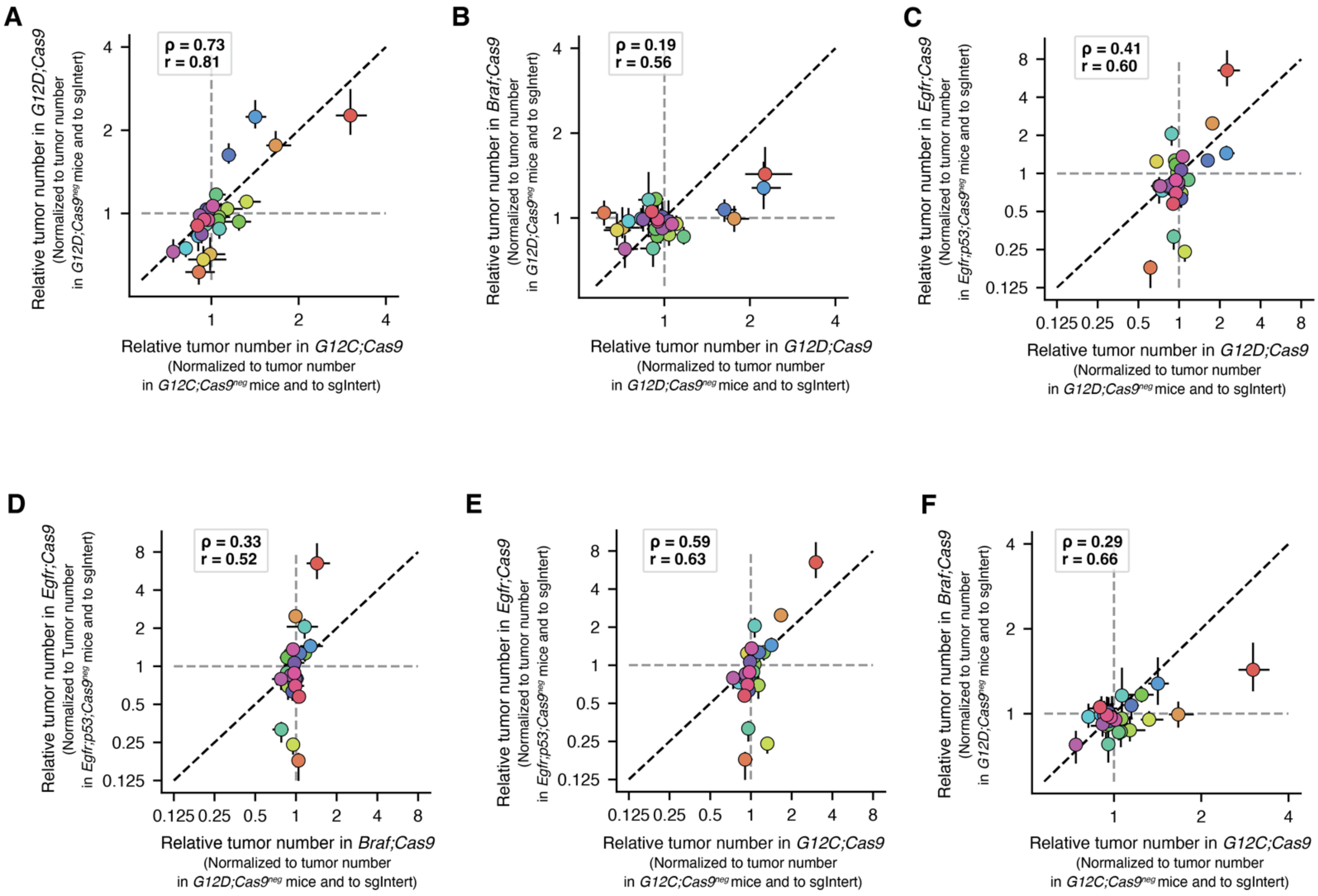

Supplementary Figure 10. Relative tumor number measurements vary across oncogenes. **A-F,** Relative tumor number in indicated genotypes of mice at 15 weeks after tumor initiation. Each dot represents the tumors initiated from one Lenti-sgRNA/Cre vector and the bars are the 95th percent confidence intervals. The black dotted line indicates equal effect. Spearman rank-order correlation (ρ) and Pearson correlation (r) are indicated.

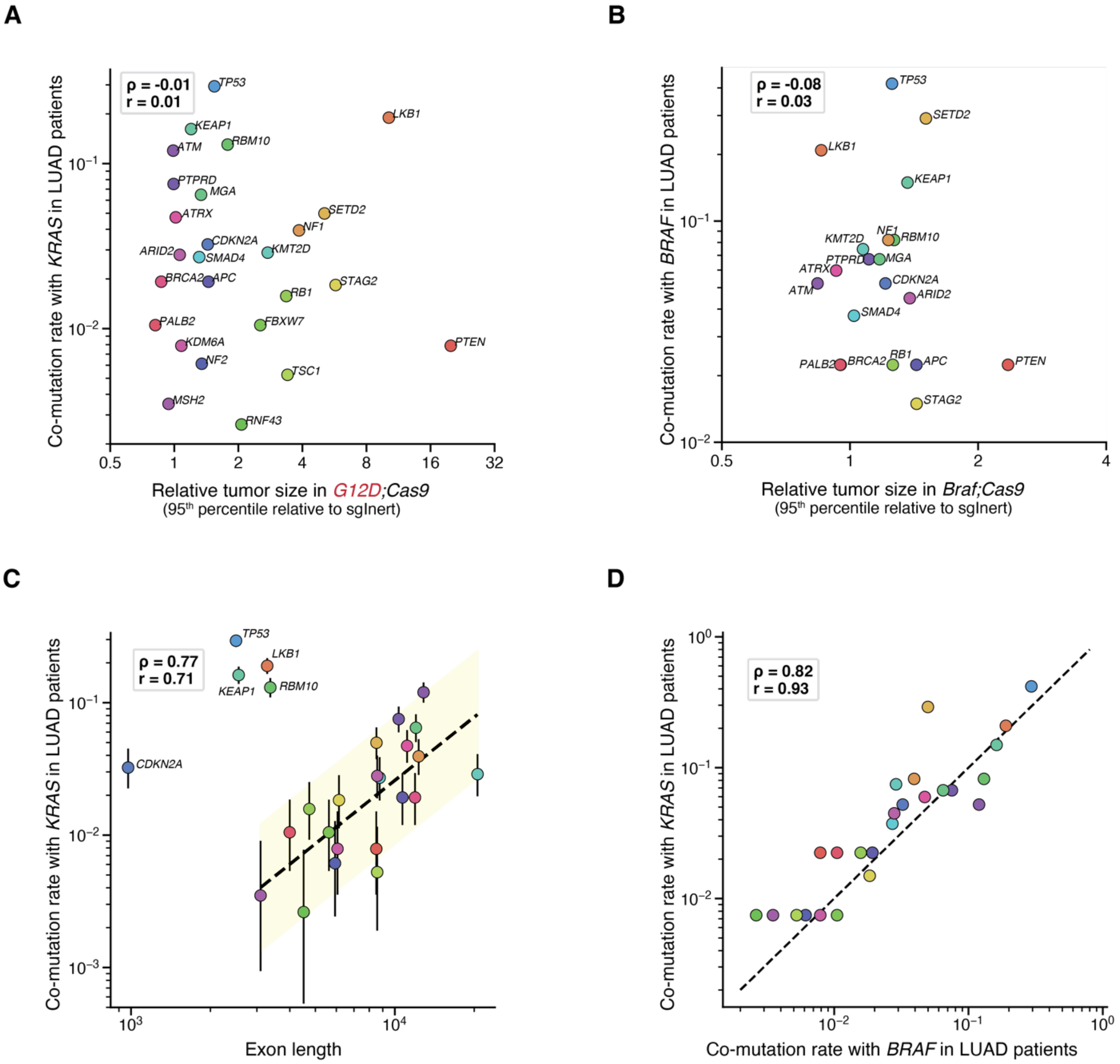

Supplementary Figure 11. Co-mutation rates in KRAS- and BRAF-driven lung adenocarcinoma are more correlated with exon length than causal effects, suggesting human frequencies in these contexts are driven primarily by passenger mutations. **A** and **B,** Correlation of relative tumor size at the 95th percentile to co-mutation rate of each gene tested in our model with *KRAS* (**A**) and *BRAF* (**B**) in LUAD patients. *CMTR2* was the only gene tested in our model that was not present in the MSK-IMPACT468 panel and therefore not included here. Spearman rank-order correlation (ρ) and Pearson correlation (r) are indicated. **C,** Correlation of exon length to co-mutation rate of each gene tested in our model with *KRAS* in LUAD patients. Error bars show the 95% binomial confidence interval. *TP53*, *LKB1*, *KEAP1*, *RBM10*, and *CDKN2A* were determined to be outliers. After removal of outliers: dotted line shows linear fit to the log-transformed exon length and log-transformed co-mutation rate; yellow region highlights 3x on either size of the fit, spearman rank-order correlation (ρ) and Pearson correlation (r) are indicated. **D,** Correlation of co-mutation rate with *BRAF* in LUAD with co-mutation rate with *KRAS* in LUAD patients. Spearman rank-order correlation (ρ) and Pearson correlation (r) are indicated.

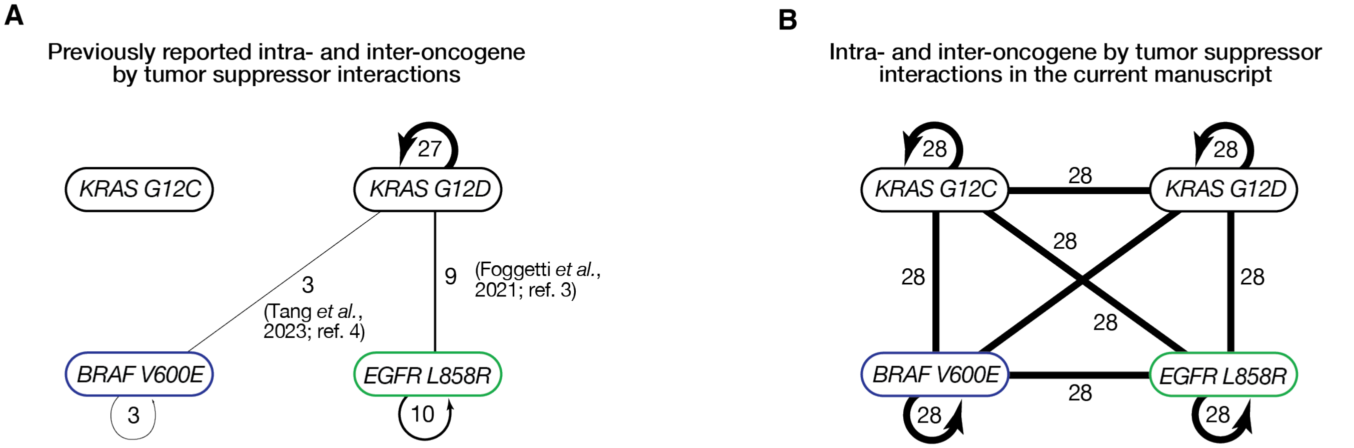

Supplementary Figure 12. Extent of novel intra- and inter-oncogene comparisons of tumor suppressor effects described in the current manuscript. **A,** Previous data investigating the impact of inactivating putative tumor suppressor genes on tumor growth within and across oncogenic contexts in quantitative *in vivo* models[3, 4]. **B,** Data from the present study investigating the impact of inactivating putative tumor suppressor genes on tumor growth within and across oncogenic contexts.

REFERENCES

1. Rogers ZN*, et al.* A quantitative and multiplexed approach to uncover the fitness landscape of tumor suppression in vivo. *Nature methods* **14**, 737-742 (2017).

2. Cai H*, et al.* A functional taxonomy of tumor suppression in oncogenic KRAS-driven lung cancer. *Cancer discovery* **11**, 1754-1773 (2021).

3. Foggetti G*, et al.* Genetic determinants of EGFR-driven lung cancer growth and therapeutic response in vivo. *Cancer discovery* **11**, 1736-1753 (2021).

4. Tang R*, et al.* Multiplexed screens identify RAS paralogues HRAS and NRAS as suppressors of KRAS-driven lung cancer growth. *Nat Cell Biol* **25**, 159-169 (2023).
