# Supplementary material for: Oncogenic context shapes the fitness landscape of tumor suppression
Source: Nat Commun. 2023 Oct 12;14:6422. doi: 10.1038/s41467-023-42156-y (PMC10570323; doi:10.1038/s41467-023-42156-y)
Supplement: Supplementary file 2 — Reporting Summary [file 41467_2023_42156_MOESM2_ESM.pdf]

nature portfolio

Corresponding author(s): Ian P. Winters, Michael J. Rosen

Last updated by author(s): Sep 7, 2023

# Reporting Summary

Nature Portfolio wishes to improve the reproducibility of the work that we publish. This form provides structure for consistency and transparency in reporting. For further information on Nature Portfolio policies, see our Editorial Policies and the Editorial Policy Checklist.

## Statistics

For all statistical analyses, confirm that the following items are present in the figure legend, table legend, main text, or Methods section.

| n/a | Confirmed | |
|---|---|---|
| ☐ | ☒ | The exact sample size ($n$) for each experimental group/condition, given as a discrete number and unit of measurement |
| ☐ | ☒ | A statement on whether measurements were taken from distinct samples or whether the same sample was measured repeatedly |
| ☐ | ☒ | The statistical test(s) used AND whether they are one- or two-sided<br>*Only common tests should be described solely by name; describe more complex techniques in the Methods section.* |
| ☒ | ☐ | A description of all covariates tested |
| ☐ | ☒ | A description of any assumptions or corrections, such as tests of normality and adjustment for multiple comparisons |
| ☐ | ☒ | A full description of the statistical parameters including central tendency (e.g. means) or other basic estimates (e.g. regression coefficient) AND variation (e.g. standard deviation) or associated estimates of uncertainty (e.g. confidence intervals) |
| ☐ | ☒ | For null hypothesis testing, the test statistic (e.g. $F$, $t$, $r$) with confidence intervals, effect sizes, degrees of freedom and $P$ value noted<br>*Give P values as exact values whenever suitable.* |
| ☒ | ☐ | For Bayesian analysis, information on the choice of priors and Markov chain Monte Carlo settings |
| ☒ | ☐ | For hierarchical and complex designs, identification of the appropriate level for tests and full reporting of outcomes |
| ☐ | ☒ | Estimates of effect sizes (e.g. Cohen's $d$, Pearson's $r$), indicating how they were calculated |

*Our web collection on statistics for biologists contains articles on many of the points above.*

## Software and code

Policy information about availability of computer code

| | |
|---|---|
| Data collection | No software was used for data collection. |
| Data analysis | The core software used is described in the Methods, under the sub-section "Analysis of sequencing data". The open-source software includes the following external tools: BCLConvert v3.8.2, CutAdapt v4.1, and Bowtie2 v2.4.4. A description of all open-source code is included in the Methods and further details are available on request. The proprietary portions of the code are not available.<br>Statistics and plots were generated using Python. Statistical tests were run using Scipy v1.8.1. |

For manuscripts utilizing custom algorithms or software that are central to the research but not yet described in published literature, software must be made available to editors and reviewers. We strongly encourage code deposition in a community repository (e.g. GitHub). See the Nature Portfolio guidelines for submitting code & software for further information.

## Data

Policy information about availability of data

All manuscripts must include a data availability statement. This statement should provide the following information, where applicable:

- Accession codes, unique identifiers, or web links for publicly available datasets
- A description of any restrictions on data availability
- For clinical datasets or third party data, please ensure that the statement adheres to our policy

The raw tumor data have been deposited in Dryad [doi:10.5061/dryad.xpnvx0kmz]. The data plotted in the main manuscript, including summary statistics and their

confidence intervals, are provided in the Source Data file.

## Human research participants

Policy information about studies involving human research participants and Sex and Gender in Research.

| | |
|---|---|
| Reporting on sex and gender | N/A |
| Population characteristics | N/A |
| Recruitment | N/A |
| Ethics oversight | N/A |

Note that full information on the approval of the study protocol must also be provided in the manuscript.

# Field-specific reporting

Please select the one below that is the best fit for your research. If you are not sure, read the appropriate sections before making your selection.

[x] Life sciences   [ ] Behavioural & social sciences   [ ] Ecological, evolutionary & environmental sciences

For a reference copy of the document with all sections, see nature.com/documents/nr-reporting-summary-flat.pdf

# Life sciences study design

All studies must disclose on these points even when the disclosure is negative.

| | |
|---|---|
| Sample size | No sample size calculation was performed for this study. However, prior studies with comparable sample sizes revealed many significant effects (Rogers et al., Nature Methods (2017); Rogers et al., Nature Genetics (2018); Cai et al., Cancer Discovery (2021); unpublished internal D2G Oncology studies). Further, our bootstrap confidence intervals capture uncertainty in statistical metrics due to random sampling of mice and tumors. |
| Data exclusions | Mice were excluded if they did not receive sufficient viral titer during transduction, as measured by tumor barcode sequencing. Although the absolute cutoff was not pre-determined, the method by which we would remove the mice (detailed in the Methods section) was determined in advance of the study. |
| Replication | Many experiments were done to reproduce our results. We replicated the effects of the tumor suppressor-targeting sgRNAs across 11 independent study groups (243 mice) in the G12D model and across 4 independent study groups (47 mice) in the G12C model. Furthermore, the effects of two independent sgRNAs targeted the same gene were tested to confirm reproducibility of the sgRNAs. See Supplementary Figure 5 for additional details and data. |
| Randomization | Mice were randomized into experimental groups within each oncogenic background, and we then confirmed that groups were comprised of approximately even sex ratios. |
| Blinding | Blinding was not relevant to our study because all mice received the same treatment and no drugs were administered. |

# Reporting for specific materials, systems and methods

We require information from authors about some types of materials, experimental systems and methods used in many studies. Here, indicate whether each material, system or method listed is relevant to your study. If you are not sure if a list item applies to your research, read the appropriate section before selecting a response.

## Materials & experimental systems

| n/a | Involved in the study | |
|---|---|---|
| [x] | [ ] | Antibodies |
| [x] | [ ] | Eukaryotic cell lines |
| [x] | [ ] | Palaeontology and archaeology |
| [ ] | [x] | Animals and other organisms |
| [x] | [ ] | Clinical data |
| [x] | [ ] | Dual use research of concern |

## Methods

| n/a | Involved in the study | |
|---|---|---|
| [x] | [ ] | ChIP-seq |
| [x] | [ ] | Flow cytometry |
| [x] | [ ] | MRI-based neuroimaging |

# Animals and other research organisms

Policy information about studies involving animals; ARRIVE guidelines recommended for reporting animal research, and Sex and Gender in Research

| | |
|---|---|
| Laboratory animals | Kras.LSL-G12D, Braf.CA-V600E, tetO-EGFR.L858R, Trp53.flox, Rosa26.LSL-rtTA3-ires-mKate, Rosa26.LSL-Cas9-2a-GFP and H11.LSL-Cas9 alleles have been described (see Methods). Mice with Kras and Braf alleles were on a BL6 (C57BL/6) background, while mice with Egfr allele were on a mixed BL6/129/FVB background. Lung tumors were initiated in 6-30 week old mice. All animals were kept in pathogen-free housing and animal experiments were conducted in accordance with protocols approved by either the Yale University Institutional Animal Care or Explora BioSciences Institutional Animal Care and Use Committee (IACUC) guidelines. Mice were housed in a pathogen-free environment in Innovive Disposable IVC cages made from 100% high-viscosity PET. Each cage had a dual HEPA-filtered ventilation system. The density of mice was limited to 5 per cage. Animal rooms had a controlled 12 hour light/dark cycle. The normal temperature and relative humidity ranges in the animal rooms were 23 ± 2.5°C and 50 ± 20%, respectively. Cages were set to have 50-60 air exchanges per hour. Water (filtered, purified, and acidified to a pH of 2.5 to 3.0; e.g. Aquavive acidified water from Innovive) and standard rodent chow (e.g. Teklad 2920X irradiated diet) were provided ad libitum. A veterinarian oversaw and maintained authority over all animal welfare. Mice experiencing pain or distress (or found moribund) as evidenced by prolonged respiratory distress, poor grooming, inability to eat, lack of movement, loss of greater than 10% of their body weight over any window of time, or a rapid or sustained deterioration in health status resulting in a Body Condition Score (BCS) of ≤ 2 were deemed to require immediate euthanasia. Mice were euthanized using CO2 followed by a secondary method (i.e. cervical dislocation or thoracotomy). |
| Wild animals | No wild animals were used in this study |
| Reporting on sex | Individual cages containing 1-5 male or female mice were assigned to study groups such that each sex was approximately evenly represented within each group |
| Field-collected samples | No field-collected samples were used in this study |
| Ethics oversight | Yale University Institutional Animal Care or Explora BioSciences Institutional Animal Care and Use Committee (IACUC) |

Note that full information on the approval of the study protocol must also be provided in the manuscript.
