# Supplementary material for: Oncogenic context shapes the fitness landscape of tumor suppression
Source: Nat Commun. 2023 Oct 12;14:6422. doi: 10.1038/s41467-023-42156-y (PMC10570323; doi:10.1038/s41467-023-42156-y)
Supplement: Supplementary file 4 — Description of Additional Supplementary Files [file 41467_2023_42156_MOESM4_ESM.pdf]

Description of Additional Supplementary Files

Supplementary Data 1
Description: Individual mouse metadata
